# Analysis of the Complete Mitochondrial Genome of *Pteronura brasiliensis* and *Lontra canadensis*

**DOI:** 10.3390/ani13203165

**Published:** 2023-10-10

**Authors:** Qinguo Wei, Xibao Wang, Yuehuan Dong, Yongquan Shang, Guolei Sun, Xiaoyang Wu, Chao Zhao, Weilai Sha, Guang Yang, Honghai Zhang

**Affiliations:** 1 Jiangsu Key Laboratory for Biodiversity and Biotechnology, College of Life Sciences, Nanjing Normal University, Nanjing 210023, China; qgwei2008@163.com (Q.W.); gyang@njnu.edu.cn (G.Y.); 2 College of Life Sciences, Qufu Normal University, Qufu 273165, China; wangxibao1995@163.com (X.W.); dongyuehuan2019@163.com (Y.D.); yongquanshang@163.com (Y.S.); sunguolei1989@163.com (G.S.); wuxiaoyang1988@126.com (X.W.); zc37130@126.com (C.Z.); shaweilai@163.com (W.S.)

**Keywords:** *Pteronura brasiliensis*, *Lontra canadensis*, mitochondrial genomes, evolution, adaptation

## Abstract

**Simple Summary:**

Mitochondria, the energy metabolism center, provide most of the energy required for life processes through oxidative phosphorylation (OXPHOS). Mitochondrial genomes, a useful type of genetic marker, are widely used in phylogenetics and evolutionary and ecological research. Herein, the full-length mitochondrial genome sequences of two otter species, *Pteronura brasiliensis* (*P. brasiliensis*) and *Lontra canadensis* (*L. canadensis*), were constructed for the first time. Comparative mitochondrial genome, selection pressure, and phylogenetic independent contrasts (PICs) analyses were employed to unveil the structure and evolutionary characteristics of their mitochondrial genomes. Additionally, phylogenetic analysis confirmed the phylogenetic positions of these otter species.

**Abstract:**

*P. brasiliensis* and *L. canadensis* are two otter species, which successfully occupied semi-aquatic habitats and diverged from other Mustelidae. Herein, the full-length mitochondrial genome sequences were constructed for these two otter species for the first time. Comparative mitochondrial genome, selection pressure, and phylogenetic independent contrasts (PICs) analyses were conducted to determine the structure and evolutionary characteristics of their mitochondrial genomes. Phylogenetic analyses were also conducted to confirm these two otter species’ phylogenetic position. The results demonstrated that the mitochondrial genome structure of *P. brasiliensis* and *L. canadensis* were consistent across Mustelidae. However, selection pressure analyses demonstrated that the evolutionary rates of mitochondrial genome protein-coding genes (PCGs) *ND1*, *ND4*, and *ND4L* were higher in otters than in terrestrial Mustelidae, whereas the evolutionary rates of *ND2*, *ND6*, and *COX1* were lower in otters. Additionally, PIC analysis demonstrated that the evolutionary rates of *ND2*, *ND4*, and *ND4L* markedly correlated with a niche type. Phylogenetic analysis showed that *P. brasiliensis* is situated at the base of the evolutionary tree of otters, and then *L. canadensis* diverged from it. This study suggests a divergent evolutionary pattern of Mustelidae mitochondrial genome PCGs, prompting the otters’ adaptation to semi-aquatic habitats.

## 1. Introduction

Otters belong to the subfamily Lutrinae within the family Mustelidae and have recently radiated from terrestrial weasel-like ancestors and successfully thrive in semi-aquatic habitats. *P. brasiliensis* and *L. canadensis* are two otter species. The giant otter (*P. brasiliensis*), which belongs to the genus *Pteronura*, is the largest and unique species of otter living in freshwater habitats [1]. The North American river otter (*L. canadensis*), belonging to the genus *Lontra*, is mainly distributed in the North American watersheds [2] and can occupy different habitats from the sea to freshwater habitats, mountain streams, and desert canyons [2]. These two species were often identified by their morphological characteristics [2,3,4]. Their phylogenetic status was also determined using their morphological characteristics or partial fragments of the mitochondrial genome [4,5,6], as their mitochondrial genomes are still unknown up to now. Therefore, we assembled the mitochondrial genomes of these two species for the first time to provide important genetic resources for future study, and to determine their phylogenetic status. Additionally, these mitochondrial genomes may be important genetic resources for protecting these two species in the future.

The mitochondrion is the center of energy metabolism [7], and its genome is independent of the nuclear genome. Mammalian mitochondrial genomes are double-stranded circular molecules approximately 16 kB nucleotide in length [8]. One strand of the mitochondrial genome is the heavy (H) strand, which is rich in guanine. The other strand, called the light (L) strand, is cytosine-rich [8,9]. The mammalian mitochondrial genome usually contains 22 transfer RNA (tRNA) genes, 13 protein-coding genes (PCGs), 2 ribosomal RNA (rRNA) genes, and a major non-coding control region (D-loop) [8,10].

The mitochondrial genome is maternally inherited; its molecular size is small, the sequencing procedures are simple, and the recombination rate is low. Therefore, they are often used to analyze phylogenetic relationships, genetic diversity, and evolutionary adaptations [11,12]. Mitochondrial genome evolution has been previously shown to be related to niche adaptation in animals. The Mustelidae mitochondrial genome had undergone divergent evolution among animals adapted to different niches [13]. The Cetartiodactyla mitochondrial genome also displayed divergent evolutionary patterns during the process of niche adaptation [14]. Positive selection signals in the mitochondrial genomes of Vesicomyidae species had revealed evidence for their adaptive to deep-sea environments [15]. Studies on the mitochondrial genome of domesticated animals, such as dogs, cattle, and yaks had shown relaxed selection patterns when compared with their wild relatives under the adaptation to domesticated environments [16,17,18]. Additionally, the mitochondrial genome of Tibetan loaches displayed more non-synonymous mutations than that of non-Tibetan loaches in order to adapt to the environment of the Tibetan plateau [19]. Based on this previous research, this study hypothesized that the mitochondrial genome of otters might have divergent evolutionary patterns in order to adapt to semi-aquatic environments when compared to their terrestrial Mustelidae close relatives.

In this study, we assembled and annotated the complete mitochondrial genome of *P. brasiliensis* and *L. canadensis* based on high-quality raw genome sequencing data for the first time to explore their structural characteristics and provide genetic resources for protecting these two important species. The mitochondrial genomes of the two genera of otters have not been previously studied. Contrastingly, in order to investigate how the otters adapt to the semi-aquatic habitats, nine otters representing all the seven genera of Lutrinae and ten Mustela who were the closest relatives of otters in Mustelidae were selected to explore the evolutionary characteristics of otters’ mitochondrial genome. Therefore, this study aimed to reveal the adaptive evolution of otters to semi-aquatic niches from the mitogenomics perspective and provide genetic resources for the protection of these two otter species.

## 2. Materials and Methods

### 2.1. Mitochondrial Genome Assembly and Annotation

High-quality raw sequencing data of otter genomes (*P. brasiliensis*: SRR12437585, *L. canadensis*: SRR10409165) were downloaded from the SRA database (https://www.ncbi.nlm.nih.gov/sra/, accessed on 30 April 2022), and then NOVOPlasty 4.1 [20] was used to assemble the two complete mitogenomes with the raw genome sequencing data and the seed sequence (EF491181.1 for *P. brasiliensis* and JF443249.1 for *L. canadensis*). Then, the assembled sequences were validated and revised with seed sequences and genome data. The validated mitochondrial genome sequences were annotated with the online software MITOS2 [21] (http://mitos2.bioinf.uni-leipzig.de/index.py, accessed on 1 June 2022). The annotation results were revised by comparing them with the mitochondrial genomes of *Enhydra lutris* (NC_009692.1) through the method of BLAST. The two mitochondrial genomes were submitted to GenBank under the accession numbers OP056177.1 (*L. canadensis*) and OP056176.1 (*P. brasiliensis*). Structure maps of these two mitochondrial genomes were drawn with the online software OGDRAW 1.3.1 [22] (https://chlorobox.mpimp-golm.mpg.de/OGDraw.html, accessed on 3 March 2023). The relative synonymous codon usage (RSCU) of the two mitochondrial genomes was calculated using MEGA X [23] and visualized with R software (V 4.1.3; package, ggplot2).

### 2.2. tRNA Gene Structure Analyses

The online software tRNAScan-SE 2.0 was used to predict the tRNA gene structure [24] (http://lowelab.ucsc.edu/tRNAscan-SE, accessed on 30 July 2022).

### 2.3. Comparative Mitochondrial Genome Analyses

The complete mitochondrial genomes of 21 Mustelidae species from 2 different habitats (terrestrial and semi-aquatic) were selected for comparison. The accession numbers of the 21 mitochondrial genomes are shown in Table S1. The nucleotide compositional bias were measured through AT skew [(A − T)/(A + T)] and GC skew [(G − C)/(G + C)] [25]. Furthermore, the synteny analysis was conducted with Mauve software (2.3.1) [26] based on the 11 mitochondrial genomes selected from the above 21 species.

### 2.4. Phylogenetic Analyses

A total of 13 different protein-coding genes (PCGs) from the 21 mitochondrial genomes were retrieved and combined into a single sequence, named 13PCG. We chose only 13PCG for phylogenetic analyses because the PCG evolutionary rate was more suitable for phylogenetic analyses than that of the other regions of the mitochondrial genome sequences. The combined sequences 13PCG were aligned with MUSCLE v3.8.31 [27]. The optimal model (GTR + G + I) was selected using the model finder function of PhyloSuite software (V 1.2.2) [28]. Bayesian inference (BI) was subsequently used to infer the phylogenetic relationships of these 21 species (Table S1). BI was conducted with MrBayes [29] using the Markov Chain Monte Carlo (MCMC) algorithm, running for 2,000,000 cycles (sampling one tree every 1000 generations). *Vormela peregusna* and *Galictis vittata* were selected as the outgroups. Using the Interactive Tree of Life (ITOL) website, the derived BI tree was visualized [30].

### 2.5. Selection Analyses

To assess the molecular evolution rate of mitochondrial genome 13 PCGs and 13PCG, we constructed 14 datasets and calculated the ratio of the non-synonymous to synonymous substitution rate (ω = dN/dS) using the codon-based maximum likelihood software (CodeML 4.9j), which was implemented in PAML 4.9j [31]. The root-to-tip ω values (average ω value from the last universal common ancestor of all species on the species tree to each terminal branch) were assessed based on the free ratio model in CodeML. Subsequently, the otters’ root-to-tip ω values were compared with those of the terrestrial Mustelidae. The Mann–Whitney–Wilcoxon test was used for this analysis. The branch model (two-ratio model, model = 2, NSsites = 0; one-ratio model, model = 0, NSsites = 0) was used to detect rapidly evolving genes in the otter branches. The tree used in this analysis was based on the traditional otter classification and the BI tree constructed above (Figure S1).

### 2.6. Phylogenetic Independent Contrast (PIC) Analysis

We performed a PIC analysis [32] on 13 PCGs and 13PCG with the *ape* package implemented in R 3.6.2 software to explore the relationship between habitat type and the dN/dS of mitochondrial genome PCGs. The tree in selection analyses was used as the input tree. The root-to-tip ω values were transformed by log10 and used as the dN/dS. According to the habitat, the otters and the other species were classified into the semi-aquatic and terrestrial groups, respectively. We subsequently coded them as 1 and 0, respectively.

## 3. Results

### 3.1. Mitochondrial Genome Structure and Annotation

**Figure 1:**
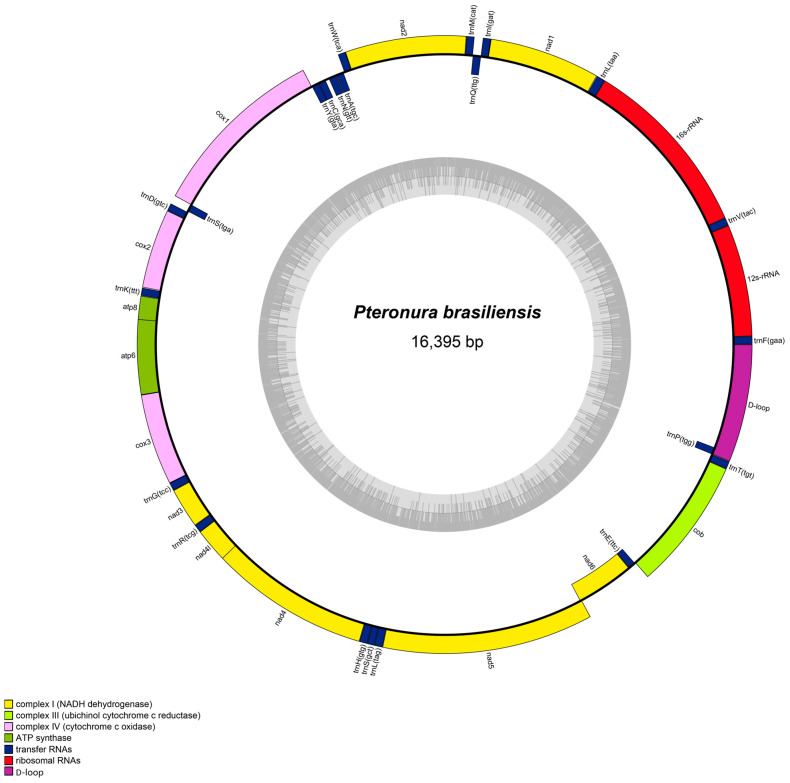
Mitochondrial genome structure map of *P. brasiliensis*. Genes encoded in the heavy strand are located on the outside of the ring, while genes encoded in the light stand are located on the inside of the ring.

The lengths of the complete mitochondrial genome of *P. brasiliensis* and *L. canadensis* were 16,395 and 16,500 bp, respectively. They both consisted of 37 genes (13 PCGs, 22 tRNA genes, and 2 rRNA genes) and a control region (D-loop) (Table 1 and Table S3; Figure 1 and Figure S2), in which 9 genes (*ND6, tRNA^GLN^, tRNA^ALA^, tRNA^ASN^, tRNA^CYS^, tRNA^TYR^, tRNA^SER2^, tRNA^GLU^*, and *tRNA^PRO^*) were distributed in the light strand, and the other 28 genes were located on the heavy strand. The initiation codon for most PCGs was ATG (number (N) = 9) and the remaining 4 were ATC, ATA, GTG, and ATT in *P. brasiliensis*, whereas the termination codons were TAA (N = 7), TAG (N = 2), TA- (N = 2), T-- (N = 1), and AGA (N = 1). Contrastingly, the common initiation codon in *L. canadensis* was also ATG (N = 9), and the remaining 4 were ATC, ATA, GTG, and ATT, while the termination codons were TAA (N = 8), TAG (N = 2), TA- (N = 1), T-- (N = 1), and AGA (N = 1). Qverlaps of 77 and 76 bp existed across the mitochondrial genomes of *P. brasiliensis* and *L. canadensis*, respectively. The longest overlap was observed between ATP8 and ATP6 (43 bp in *P. brasiliensis* and 40 bp in *L. canadensis*).

GC nucleotide proportions were 39.5% in the complete mitochondrial genome of *P. brasiliensis* and 42.8% in that of *L. canadensis*, respectively, which were lower than those of the AT nucleotide proportions. The AT skew for the complete mitochondrial genome of *P. brasiliensis* and *L. canadensis* were 0.074 and 0.094, respectively, whereas the GC skew were −0.274 and −0.274, respectively. For both of the two species, the composition proportions of AT nucleotide were also higher than that of the GC nucleotide in the PCGs, tRNAs, rRNAs, and D-loops individually. The AT skew and GC skew were slightly positive and negative, respectively (Table 2 and Table 3). This suggested that the proportion of A was higher than that of T, while the proportion of C was higher than that of G.

**Figure 2:**
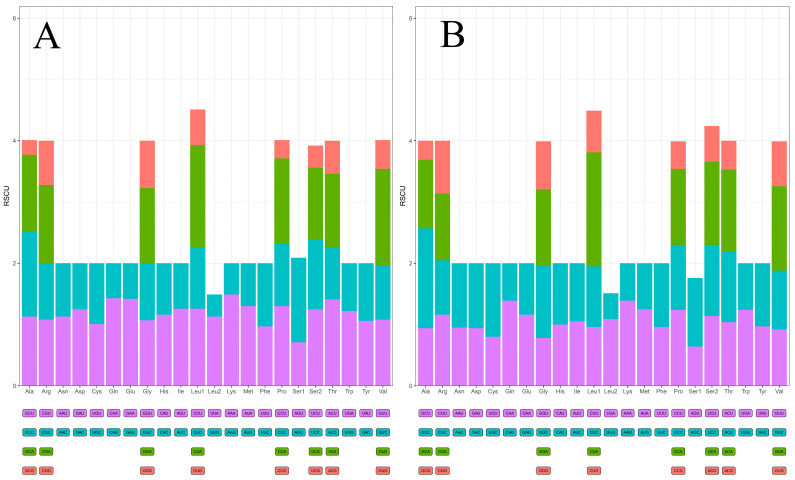
Relative synonymous codon usage (RSCU) of *P. brasiliensis* (**A**) and *L. canadensis* (**B**). The *x*-axis represents the amino acids encoded by the codon, which is listed beneath each amino acid. The RSCU values are listed on the *y*-axis.

According to the codon usage analysis, the two species exhibited a strong preference for eight codon families (Leu1, Val, Ala, Arg, Pro, Thr, Gly, and Ser2) (Figure 2).

### 3.2. tRNA Gene Structure

**Figure 3:**
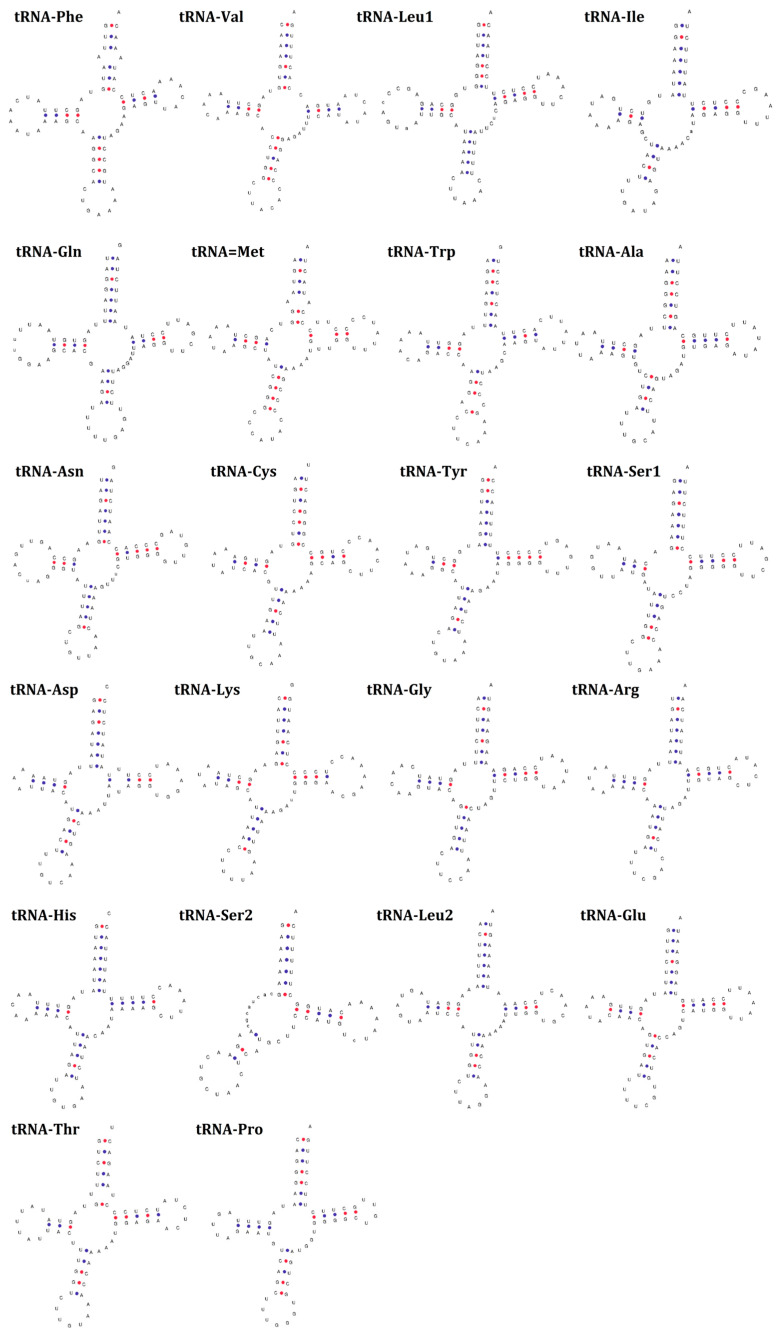
The predicted secondary structures of 22 tRNAs genes in *P. brasiliensis* mitochondrial genome.

Based on the tRNA gene sequences identified in the annotation, their secondary structures were determined using tRNAScan-SE 2.0. Except for *tRNA^SER2(GCT)^*, all of the remaining 21 tRNAs had a canonical cloverleaf structure. However, the *tRNA^SER2(GCT)^* lacked the dihydrouridine hairpin structure (Figure 3 and Figure S3).

### 3.3. Comparison of Mitochondrial Genomes among Species

The nucleotide composition of the heavy strand of the mitochondrial genome was generally consistent in otters and Mustelidae. The proportion of A was higher than that of T, whereas the proportion of G was lower than that of C. The AT skew of the mitochondrial genome’s heavy strand was slightly positive, whereas the GC skew was slightly negative. Contrastingly, the AT proportion was higher than that of GC in all of the 21 species (Table 4).

**Figure 4:**
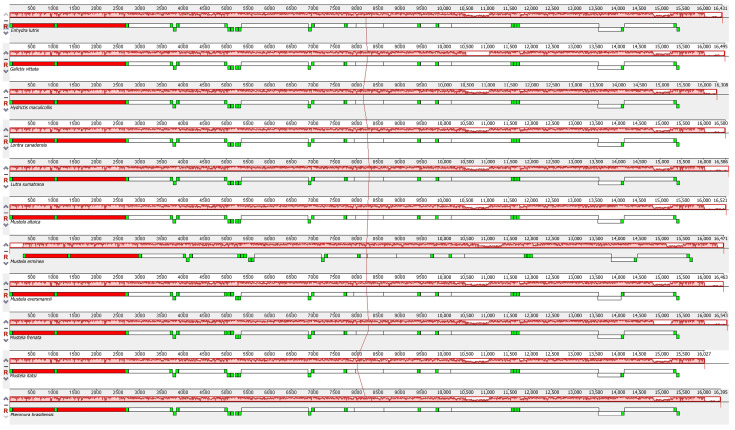
Gene arrangement comparison of the 11 species selected in this study with Mauve. PCG is in the white block, 12S rRNA and 16S rRNA are in the red block, and tRNA is in the green block.

Comparative alignment of the 11 mitochondrial genomes showed that the gene order was conserved among otters and their close Mustela relatives (Figure 4).

### 3.4. Phylogenetic Analyses

**Figure 5:**
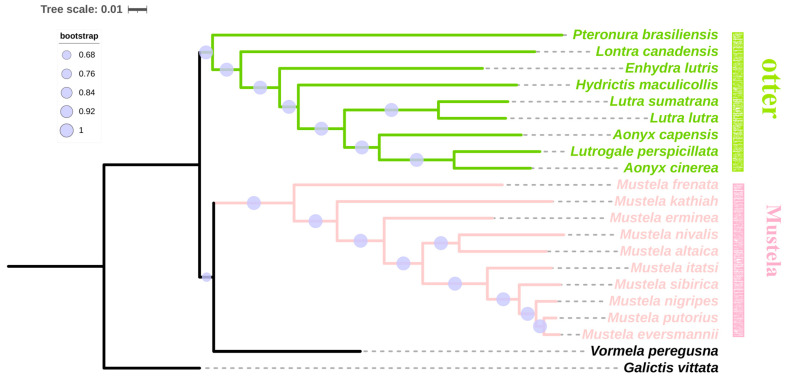
Phylogenetic relationships of 21 species evaluated in this study based on nucleotide dataset of the 13 mitochondrial protein-coding genes through the method of Bayesian inference.

All otters were clustered in one clade, whereas the Mustela species were clustered in another clade with high Bayesian posterior probabilities (Figure 5). *P. brasiliensis* was the earliest otter species to diverge from the Mustela species. Subsequently, *L. canadensis* appeared. *Lutra lutra* and *Lutra sumatrana* were clustered into one clade; *Aonyx cinerea*, *Lutrogale perspicillata*, and *Aonyx capensis* were clustered into another clade. The remaining *Enhydra lutris* and *Hydrictis maculicollis* individually formed single clades each.

### 3.5. Selection Analyses

**Figure 6:**
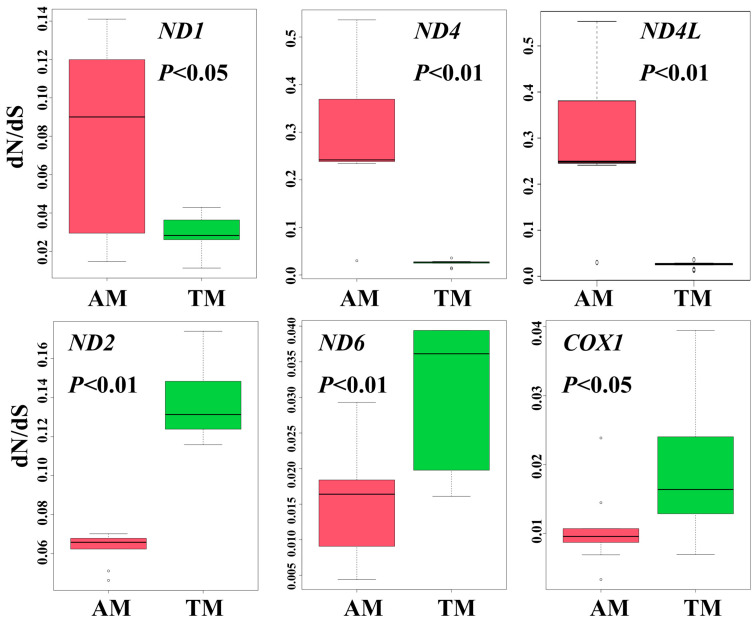
Comparisons of root-to-tip ω values among 21 selected species between otters and Mustela, based on 13 protein-coding genes (13PCG) and each PCG. The figure showed genes that have significantly different ω values. dN/dS: root to tip ω values; AM: otters, TM: Mustela.

**Figure 7:**
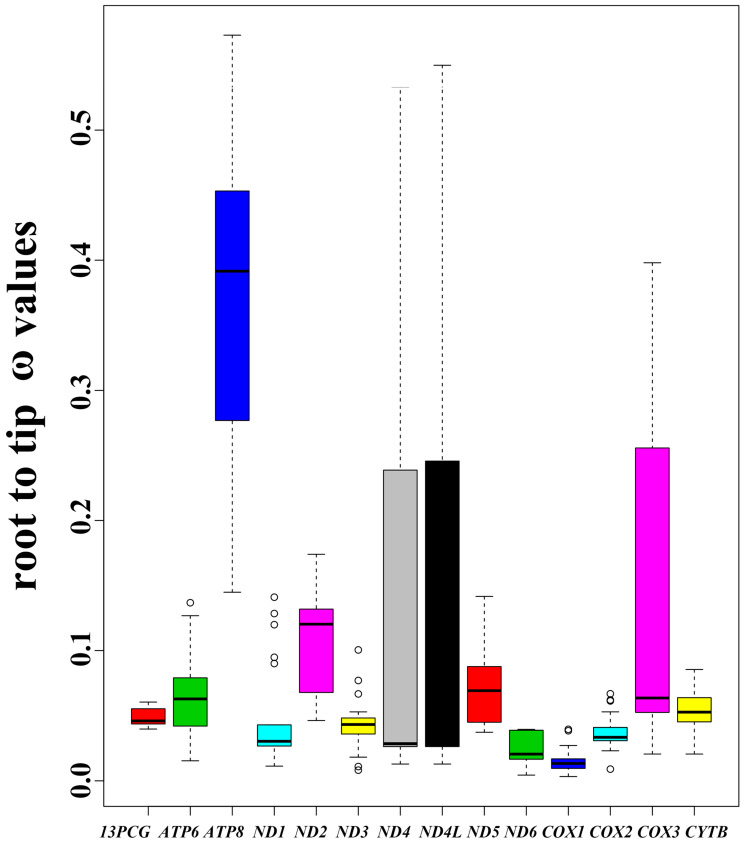
The molecular evolution rate (root-to-tip ω values) of 13 protein-coding genes (13PCG) and each PCG.

Based on the root-to-tip ω values, the mitochondrial genome PCGs of 21 Mustelidae species were mainly under purifying selection (Table S2). The result of comparing the mitochondrial genome PCGs ω values of otters with that of other Mustelidae species demonstrated that the ω values of ND1, ND4, and ND4L were higher in otters than in terrestrial Mustelidae, whereas ND2, ND6, and COX1 had lower ω values in otters (Figure 6). The result also indicated that ATP8 had the highest ω values in all of the 13 PCGs in these 21 species (Figure 7). However, the difference between otters and terrestrial Mustelidae’s ω values on ATP8 was insignificant. In the otter clade, 6 of the 13 PCGs of the mitochondrial genomes were rapidly evolving. The rapidly evolving genes were ND1, ND4, ND4L, ND5, COX3, and CYTB (Table 5). Combined with the root-to-tip ω values, we predicted that the three genes ND1, ND4, and ND4L likely evolved more quickly in otters than in terrestrial Mustelidae species.

### 3.6. PIC Analysis

**Figure 8:**
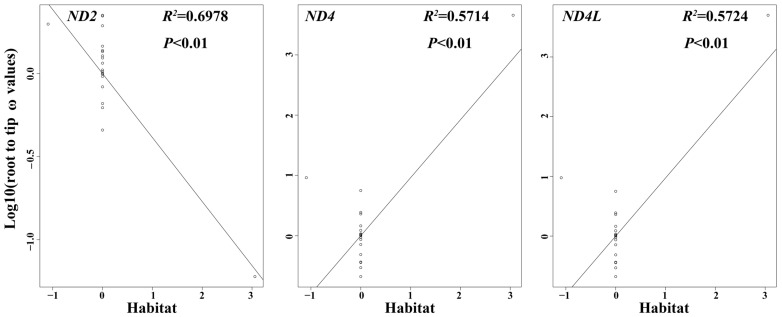
Phylogenetic independent contrast analysis between different habitats and root-to-tip ω values (Log10-transformed) on *ND2*, *ND4*, and *ND4L* in 21 selected species.

PIC analysis demonstrated that the correlation between evolutionary rates and habitats was significant for the genes *ND2*, *ND4*, and *ND4L*. This demonstrated that habitat type significantly influenced the evolutionary rate of these three genes in otters and terrestrial Mustelidae species (Figure 8).

## 4. Discussion

Mitochondria are the key cellular organs that provide energy for the life activities of animals through OXPHOS [33,34,35]. The mammalian mitochondrial genome is independent of the nuclear genome and is a double-stranded circular molecule, approximately 16 kb in length [8]. One strand of the guanine-rich mitochondrial genome is called the heavy (H) strand, and the other cytosine-rich strand is called the light (L) strand [8,9]. The mitochondrial genome is maternally inherited and often used to analyze phylogenetic relationships and evolutionary adaptations [11,12]. Additionally, it is extremely important in conservation genetics research. Therefore, we assembled two mitochondrial genomes for the two otter species that needed to be protected and studied.

Characteristics of the mitochondrial genome may differ among different animal groups. Herein, the mitochondrial genomes of *P. brasiliensis* and *L. canadensis* were 16,395 and 16,500 bp, respectively, in length and contained 37 genes (13 PCGs, 22 tRNA genes, and 2 rRNA genes) and a control region (D-loop). Among these, 28 genes were in the heavy strand and 9 other genes were in the light strand. The initiation codon for most PCGs was ATG, and the common termination codon was TAA. These characteristics were consistent with those of the mitochondrial genome’s characteristics of other otters [36,37,38,39,40]. The overall AT content of the heavy strands of these two mitochondrial genomes was higher than the GC content, indicating AT-rich characteristics. The base compositions were skewed similarly to those of other vertebrate mitochondrial genome sequences [41,42,43]. The AT content was higher than the GC content in most of the mitochondrial genomes of otters (Table 4). In bacteria, the GC skew represents the footprint of genome evolution driven by DNA replication [44]. Whether there was a relationship between the GC skew of the mitochondrial genomes and the otter species evolution remains unknown.

Among the 22 tRNAs, the *tRNA^SER2(GCT)^* lacked the dihydrouridine hairpin structure, and all the remaining 21 tRNAs had a canonical cloverleaf structure. The *tRNA^SER^* was found to lack a canonical cloverleaf structure in several animals [37,40,42,45]. Several studies have demonstrated that the lack of a dihydrouridine arm or thymidine-pseudouridine-cytidine (TψC) loop in *tRNA^SER^* might not affect its normal function [46,47]. This suggested that *tRNA^SER2(GCT)^* was able to perform normal functions in these two otter species.

The results of the phylogenetic analysis showed that all otters were clustered into one clade, whereas the Mustela species were clustered in another clade. *P. brasiliensis* was the earliest otter species to diverge from the Mustela species, which formed the genus *Pteronura*. Subsequently, *L. canadensis* branched out from *P. brasiliensis*. *E. lutris* and *H. maculicollis* individually formed one single clade each, followed by *L. canadensis*, which formed the genera *Enhydra* and *Hydrictis*. *L. lutra* and *L. sumatrana* clustered into one clade, belonging to the genus *Lutra*. These evolutionary relationships were consistent with the results of previous traditional studies on otter classification [48]. *L. perspicillata*, *A. cinerea*, and *A. capensis* were clustered into one clade. Several previous studies had demonstrated that *L. perspicillata* and *A. cinerea* clustered into one clade [39,49,50,51,52,53], which was inconsistent with the traditional classification [48]. We inferred that this might be the result of hybridization between *L. perspicillata* and *A. cinerea* [49].

Otters are semi-aquatic mammals, with important characteristics that distinguish them from other terrestrial Mustelidae species. Habitat and locomotive styles have been shown to exert a certain influence on the evolution of animal mitochondrial genomes. For example, ecological specialization exerted selective constraints on the mitochondrial genomes of Mustelidae [13]. The mitochondrial genome of some domesticated lineages, such as dogs, cattle, yaks, pigs, and silkworms, and weakly locomotive mollusks, birds, and mammalian lineages showed a relaxed evolutionary selection pattern [16,17,18,54,55,56,57]. Different habitats and lifestyles affect the evolutionary style of fish mitochondrial genomes [43,58]. Additionally, the evolution of the Cetartiodactyla mitochondrial genome displayed divergent patterns during the process of niche adaptation [14]. We analyzed the evolutionary patterns of the otters’ mitochondrial genomes through the method of comparative mitogenomics to clarify the influence of semi-aquatic habitats. The result of root-to-tip ω values demonstrated that the mitochondrial genome PCGs of the nine otters were mainly under purifying selection. This was consistent with the results of previous studies in other animals [59]. Furthermore, the ω values of *ND1*, *ND4*, and *ND4L* were higher in otters than in terrestrial Mustelidae, whereas *ND2*, *ND6*, and *COX1* had lower ω values in otters. Additionally, the studies on Tibetan loaches found some differences in mitochondrial genome PCGs’ ω values when compared with the plain species [19]. Our results also showed that *ATP8* had the highest ω values in all of the 13 PCGs in these 21 species. *ATP8* is suggested to have a high evolutionary rate in several animals [13,14,56]. *ATP8* plays an important role in metabolism, respiratory electron transport, and heat production [60]. The high evolutionary rate of *ATP8* in animals may allow for several further beneficial substitutions, which may be advantageous for animals to adapt to different ecological niches [61,62]. Contrastingly, six (*ND1*, *ND4*, *ND4L*, *ND5*, *COX3*, and *CYTB*) of the thirteen PCGs of the mitochondrial genome were rapidly evolving in the otter branch. This suggested that the otter group accumulated more nonsynonymous mutations than other terrestrial Mustelidae animals in these six genes. The high number of nonsynonymous mutations might result in a few beneficial amino acid changes, which may help otters adapt to semi-aquatic habitats [19,61,62,63]. Studies on galliform birds and loaches found that high-altitude species had large dN/dS for the 13 concatenated mitochondrial PCGs [19,64] because of their high energy demands. Combined with the root-to-tip ω values, we predict that the three genes (*ND1*, *ND4*, and *ND4L*) likely evolved more quickly in otters than in terrestrial Mustelidae species. We inferred that these rapidly evolving genes are related to otters adapting to semi-aquatic habitats.

We conducted a PIC analysis to eliminate the impact of evolutionary relationships on the mitochondrial genome evolution. A significant correlation between evolutionary rates and habitats for *ND2*, *ND4*, and *ND4L* was observed. This suggested that habitat type markedly influenced the evolutionary rate of these three genes in otters and terrestrial Mustelidae species, and mitochondrial gene evolution in otters might be correlated with their adaptation to semi-aquatic habitats.

## 5. Conclusions

The mitochondrial genomes of *P. brasiliensis* and *L. canadensis* were constructed for the first time, which will be helpful for the protection of these two otter species in the future. The structural characteristics of these two mitochondrial genomes are consistent with those of other otter species. Selective pressure and PIC analyses demonstrated that habitat type markedly influenced the evolutionary rate of otter PCGs of mitochondrial genomes and the evolution of mitochondrial genomes in otters might be correlated with their adaptation to semi-aquatic habitats.

## Figures and Tables

**Table 1:** Characteristics of the mitochondrial genome of *P. brasiliensis*.

Gene	Nucleotide Positions	Size (bp)	Stand	Intergenic Nucleotide	Start	Stop
*tRNA^PHE^*	1–69	69	+			
*12s rRNA*	72–1033	962	+	2		
*tRNA^VAL^*	1034–1101	68	+	0		
*16s rRNA*	1102–2670	1569	+	0		
*tRNA^LEU^*	2671–2745	75	+	0		
*ND1*	2748–3704	957	+	2	ATG	TAG
*tRNA^ILE^*	3704–3772	69	+	−1		
*tRNA^GLN^*	3770–3843	74	−	−3		
*tRNA^MET^*	3845–3913	69	+	1		
*ND2*	3914–4957	1044	+	0	ATC	TAG
*tRNA^TRP^*	4956–5023	68	+	−2		
*tRNA^ALA^*	5033–5101	69	−	9		
*tRNA^ASN^*	5103–5175	73	−	1		
*tRNA^CYS^*	5209–5275	67	−	33		
*tRNA^TYR^*	5276–5343	68	−	0		
*COX1*	5345–6889	1545	+	1	ATG	TAA
*tRNA^SER^*	6887–6955	69	−	−3		
*tRNA^ASP^*	6962–7028	67	+	6		
*COX2*	7029–7712	684	+	0	ATG	TAA
*tRNA^LYS^*	7716–7783	68	+	3		
*ATP8*	7785–7988	204	+	1	ATG	TAA
*ATP6*	7946–8626	681	+	−43	ATG	TAA
*COX3*	8626–9410	785	+	−1	ATG	TA-
*tRNA^GLY^*	9410–9479	70	+	−1		
*ND3*	9480–9827	348	+	0	ATA	TAA
*tRNA^ARG^*	9828–9895	69	+	0		
*ND4L*	9896–10,192	297	+	0	GTG	TAA
*ND4*	10,186–11,563	1378	+	−7	ATG	T--
*tRNA^HIS^*	11,564–11,632	69	+	0		
*tRNA^SER^*	11,633–11,694	62	+	0		
*tRNA^LEU^*	11,695–11,764	70	+	0		
*ND5*	11,765–13,585	1821	+	0	ATT	TAA
*ND6*	13,570–14,102	533	−	−16	ATG	TA-
*tRNA^GLU^*	14,103–14,171	69	−	0		
*CYTB*	14,176–15,315	1140	+	4	ATG	AGA
*tRNA^THR^*	15,316–15,383	68	+	0		
*tRNA^PRO^*	15,384–15,449	66	−	0		

**Table 2:** Nucleotide composition and AT/GC skew of the *P. brasiliensis* mitochondrial genome.

*P. brasiliensis*	Size	A%	T%	G%	C%	AT%	GC%	AT Skew	GC Skew
mtDNA	16,395.0	32.5	28.0	14.3	25.2	60.5	39.5	0.074	−0.276
PCGs	11,414.0	30.6	29.7	14.0	25.6	60.3	39.7	0.015	−0.293
tRNAs	1515.0	33.1	31.2	18.5	17.2	64.3	35.7	0.030	0.036
rRNAs	2532.0	36.1	24.0	18.2	21.7	60.1	39.9	0.201	−0.088
D-loop	1010.0	29.7	27.9	15.5	26.8	57.6	42.4	0.031	−0.267

**Table 3:** Nucleotide composition and AT/GC skew of the *L. canadensis* mitochondrial genome.

*L. canadensis*	Size	A%	T%	G%	C%	AT%	GC%	AT Skew	GC Skew
mitogenome	16,500.0	31.3	25.9	15.5	27.2	57.2	42.7	0.094	−0.274
PCGs	11,412.0	29.0	27.4	15.5	28.1	56.4	43.6	0.028	−0.289
tRNAs	1512.0	31.9	30.6	19.7	17.8	62.5	37.5	0.021	0.051
rRNAs	2530.0	36.0	22.8	18.5	22.6	58.8	41.1	0.224	−0.100
D-loop	1123.0	30.0	26.0	16.4	27.6	56	44	0.071	−0.255

**Table 4:** Nucleotide composition and AT/GC skew of 21 species mitogenomes.

Species	T(U)%	A%	AT%	AT Skew	C%	G%	GC%	GC Skew
*Pteronura brasiliensis*	28.0	32.5	60.5	0.074	25.2	14.3	39.5	−0.276
*Lontra canadensis*	25.9	31.3	57.2	0.094	27.2	15.5	42.8	−0.274
*Hydrictis maculicollis*	26.9	31.8	58.7	0.083	26.4	14.8	41.3	−0.281
*Aonyx cinerea*	25.3	31.7	57.0	0.112	28.0	15.0	43.0	−0.303
*Aonyx capensis*	25.6	31.7	57.3	0.107	27.9	14.8	42.7	−0.307
*Enhydra lutris*	26.4	32.5	58.9	0.104	26.9	14.2	41.1	−0.308
*Lutra lutra*	25.8	32.3	58.1	0.112	27.5	14.4	41.9	−0.313
*Lutrogale perspicillata*	25.5	31.1	56.6	0.100	28.0	15.4	43.4	−0.289
*Lutra sumatrana*	25.8	32.6	58.3	0.116	27.5	14.2	41.7	−0.318
*Mustela frenata*	27.4	33.3	60.8	0.096	25.8	13.5	39.2	−0.314
*Mustela eversmannii*	27.3	32.8	60.0	0.091	26.1	13.9	40.0	−0.305
*Mustela itatsi*	27.5	33.0	60.5	0.091	25.7	13.7	39.5	−0.304
*Mustela nigripes*	27.2	32.9	60.1	0.095	26.2	13.8	39.9	−0.310
*Mustela putorius*	27.4	32.8	60.2	0.091	26.0	13.8	39.8	−0.308
*Mustela erminea*	26.6	33.4	60.1	0.113	26.5	13.4	39.9	−0.327
*Mustela kathiah*	27.9	33.3	61.1	0.088	25.3	13.6	38.9	−0.301
*Mustela nivalis*	27.3	32.6	60.0	0.088	26.0	14.0	40.0	−0.299
*Mustela sibirica*	27.3	32.9	60.2	0.093	26.0	13.9	39.8	−0.304
*Mustela altaica*	27.6	32.8	60.3	0.087	25.8	13.9	39.7	−0.301
*Vormela peregusna*	27.6	33.4	61.0	0.095	26.1	12.9	39.0	−0.338
*Galictis vittata*	26.8	32.3	59.2	0.093	26.3	14.5	40.8	−0.290

**Table 5:** The rapid evolution genes in otters’ branches (bold character in the table).

Gene	Omega Background	Omega Forward Branches	2ΔlnL	*p* Value
***ND1***	**0.0147**	**0.0233**	**4.2566**	**0.0391**
***ND4***	**0.0226**	**0.0355**	**7.6079**	**<0.01**
***ND4L***	**0.0226**	**0.0355**	**7.6079**	**<0.01**
***ND5***	**0.0491**	**0.0628**	**4.0296**	**0.0447**
***COX3***	**0.0253**	**0.0493**	**15.3550**	**<0.01**
***CYTB***	**0.0253**	**0.0493**	**15.3550**	**<0.01**
*ND2*	0.0632	0.0665	0.1480	0.7004
*ND3*	0.0332	0.0459	1.3190	0.2508
*ND6*	0.0182	0.0264	1.5611	0.2115
*COX1*	0.0121	0.0085	1.9831	0.1591
*COX2*	0.0145	0.0173	0.3077	0.5791
*ATP6*	0.0338	0.0506	3.4832	0.0620
*ATP8*	0.1867	0.2720	1.6266	0.2022

## Data Availability

All the mitochondria genome sequences used in this study were downloaded from the GenBank database using the accession numbers in Table S1.

## Supplementary Materials

The following supporting information can be downloaded at https://www.mdpi.com/article/10.3390/ani13203165/s1. Table S1: The twenty-one species information selected in this study. Table S2: The root-to-tip ω values of mitochondrial genomes 13PCG and each PCG of twenty-one species. Table S3: Characteristics of the mitochondrial genome of *L. canadensis*. Figure S1: The tree used in the selection and PIC analyses. Figure S2: Mitochondrial genome structure map of *L. canadensis*. Figure S3: The predicted secondary structures of 22 tRNAs genes in *L. canadensis* mitochondrial genome.
