# Supplementary material for: Analysis of the Complete Mitochondrial Genome of Pteronura brasiliensis and Lontra canadensis
Source: Animals (Basel). 2023 Oct 10;13(20):3165. doi: 10.3390/ani13203165 (PMC10603698; doi:10.3390/ani13203165)
Supplement: Supplementary file 1 [file animals-13-03165-s001.zip › animals-2543610-supplementary.pdf]

Table S1 Species selected in this study

Species	abbreviation	GeneBank NO.
Pteronura brasiliensis	Pbra	OP056176.1 This study
Lontra canadensis	Lcan	OP056177.1 This study
Hydrictis maculicollis	Hmac	NC_046485.1
Aonyx cinerea	Acin	NC_035814.1
Aonyx capensis	Acap	NC_046484.1
Enhydra lutris	Eken	NC_009692.1
Lutra lutra	Llut	NC_062277.1
Lutrogale perspicillata	Lper	NC_035811.1
Lutra sumatrana	Lsum	NC_035810.1
Mustela frenata	Mfre	NC_020640.1
Mustela eversmannii	Meve	NC_028013.1
Mustela itatsi	Mita	NC_034330.1
Mustela nigripes	Mnig	NC_024942.1
Mustela putorius	Mfur	NC_020638.1
Mustela erminea	Merm	NC_025516.1
Mustela kathiah	Mkat	NC_023210.1
Mustela nivalis	Mniv	NC_020639.1
Mustela sibirica	Msib	NC_020637.1
Mustela altaica	Malt	NC_021751.1
Vormela peregusna	Vper	NC_054246.1
Galictis vittata	Gvit	NC_053973.1

Table S2 root to tip ω values

gene	Mfur	Meve	Mnig	Msib	Mita	Mniv	Malt	Merm	Mkat	Mfre	Vper	Gvit	Pbra	Lcan	Eken	Hmac	Lsum	Llut	Lper	Acin	Acap
ND1	0.0429	0.038967	0.0425	0.027833	0.025433	0.0287	0.03375	0.02675	0.0279	0.0243	0.0333	0.0113	0.0147	0.0178	0.03025	0.0295	0.094825	0.09005	0.128467	0.141033	0.1199
COX2	0.033333	0.0613	0.033333	0.041475	0.030733	0.03305	0.03305	0.0309	0.030467	0.0529	0.009	0.0231	0.0295	0.0307	NA	0.0668	0.04035	0.04035	0.062	0.0395	0.0395
COX3	0.072533	0.085333	0.074	0.06442	0.06345	0.052575	0.052475	0.063533	0.0531	0.04505	0.0206	0.028	0.0302	0.0546	0.041	0.398	0.25576	0.2731	0.25792	0.25984	0.25984
ATP6	0.136786	0.126814	0.11555	0.0738	0.078883	0.0606	0.07432	0.056925	0.066833	0.04195	0.0327	0.0154	0.0423	0.0393	0.02805	0.02105	0.062533	0.062825	0.07905	0.0918	0.0918
ND4L	0.028214	0.024933	0.026343	0.035757	0.028467	0.02725	0.026	0.024625	0.0129	0.0149	0.0269	0.02735	0.0299	0.55335	0.382667	0.380833	0.24528	0.24108	0.24566	0.24868	0.24868
ATP8	0.3928	0.391533	0.391533	0.339325	0.4282	0.4678	0.362925	0.30515	0.33425	0.4814	0.2034	0.2421	0.274	0.2769	0.14485	0.199833	0.57665	0.52546	0.50035	0.45318	0.45318
CYTB	0.07265	0.08545	0.07418	0.06458	0.06365	0.052775	0.052675	0.0638	0.05325	0.0452	0.0206	0.0279	0.0302	0.0544	0.0403	0.04755	0.045075	0.0667	0.047625	0.050025	0.050025
ND3	0.043167	0.043167	0.066675	0.048175	0.049867	0.036975	0.045033	0.0082	0.011	0.0367	0.0295	0.0402	0.1005	0.0182	0.03585	0.0383	0.02955	0.052875	0.077	0.0445	0.0445
COX1	0.03946	0.03836	0.016667	0.027175	0.013367	0.012	0.0154	0.0209	0.0169	0.01235	0.00695	0.0161	0.0033	0.0069	0.0087	0.0089	0.009567	0.014467	0.023867	0.0107	0.0107
ND6	0.0394	0.0394	0.0394	0.0394	0.0394	0.034133	0.0303	0.01685	0.0161	0.02055	0.019	0.0381	NA	0.0101	0.0164	NA	0.0044	0.00805	0.0293	0.0184	0.0184
ND2	0.124975	0.1379	0.130838	0.131886	0.15895	0.115917	0.128717	0.12275	0.1203	0.135567	0.16895	0.174	0.0678	0.0511	0.06855	0.046233	0.06532	0.0658	0.06234	0.06786	0.07018
ND4	0.028214	0.024933	0.026343	0.035757	0.028467	0.02725	0.026	0.024625	0.0129	0.0149	0.0269	0.02735	0.0299	0.53605	0.371133	0.3693	0.23836	0.23416	0.23874	0.24176	0.24176
ND5	0.075425	0.069163	0.070813	0.063643	0.037767	0.037183	0.03855	0.0521	0.040067	0.03735	0.14165	0.1405	0.067	0.0513	0.0449	0.098375	0.100517	0.083367	0.098367	0.0877	0.0877
13PCG	0.045711	0.048778	0.047363	0.0499	0.044117	0.039683	0.040117	0.04375	0.0436	0.04465	0.05565	0.05865	0.0422	0.04185	0.043333	0.045925	0.0535	0.055267	0.060417	0.058	0.056

Table S3 Characteristics of the mitochondrial genome of *L. canadensis*

Gene	Nucleotide Positions	Size (bp)	Stand	Intergenic Nucleotide	Start	Stop
tRNA^{PHE}	1-69	69	+			
12s rRNA	70-1033	964	+	0		
tRNA^{VAL}	1034-1100	67	+	0		
16s rRNA	1101-2666	1566	+	0		
tRNA^{LEU}	2667-2741	75	+	0		
ND1	2744-3700	957	+	2	ATG	TAG
tRNA^{ILE}	3700-3768	69	+	−1		
tRNA^{GLN}	3766-3839	74	−	−3		
tRNA^{MET}	3841-3909	69	+	1		
ND2	3910-4953	1044	+	0	ATC	TAG
tRNA^{TRP}	4952-5018	67	+	−2		
tRNA^{ALA}	5028-5096	69	−	9		
tRNA^{ASN}	5098-5170	73	−	1		
tRNA^{CYS}	5204-5270	67	−	33		
tRNA^{TYR}	5271-5338	68	−	0		
COX1	5340-6884	1545	+	1	ATG	TAA
tRNA^{SER}	6882-6950	69	−	−3		
tRNA^{ASP}	6957-7023	67	+	6		
COX2	7024-7707	684	+	0	ATG	TAA
tRNA^{LYS}	7711-7777	68	+	3		
ATP8	7779-7979	201	+	1	ATG	TAA
ATP6	7940-8620	681	+	−40	ATG	TAA
COX3	8620-9404	785	+	−1	ATG	TA-
tRNA^{GLY}	9404-9473	70	+	−1		
ND3	9474-9821	348	+	0	ATA	TAA
tRNA^{ARG}	9822-9889	68	+	0		
ND4L	9890-10186	297	+	0	GTG	TAA
ND4	10180-11557	1378	+	−7	ATG	T--
tRNA^{HIS}	11558-11626	69	+	0		
tRNA^{SER}	11627-11688	62	+	0		
tRNA^{LEU}	11689-11758	70	+	0		
ND5	11759-13579	1821	+	0	ATT	TAA
ND6	13563-14096	534	−	−17	ATG	TAA
tRNA^{GLU}	14097-14165	69	−	0		
CYTB	14170-15309	1140	+	4	ATG	AGA
tRNA^{THR}	15310-15377	68	+	0		
tRNA^{PRO}	15377-15442	66	−	−1		

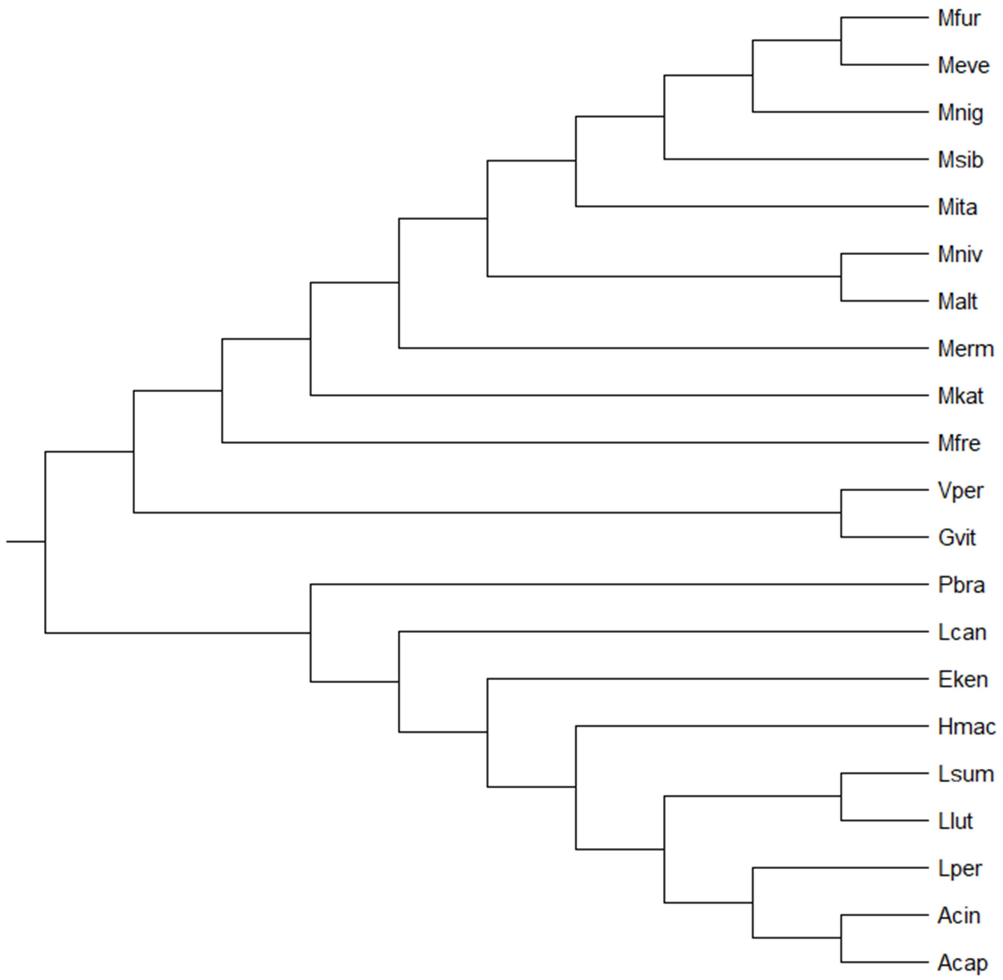

Figure S1 The tree used in the selection and PIC analyses

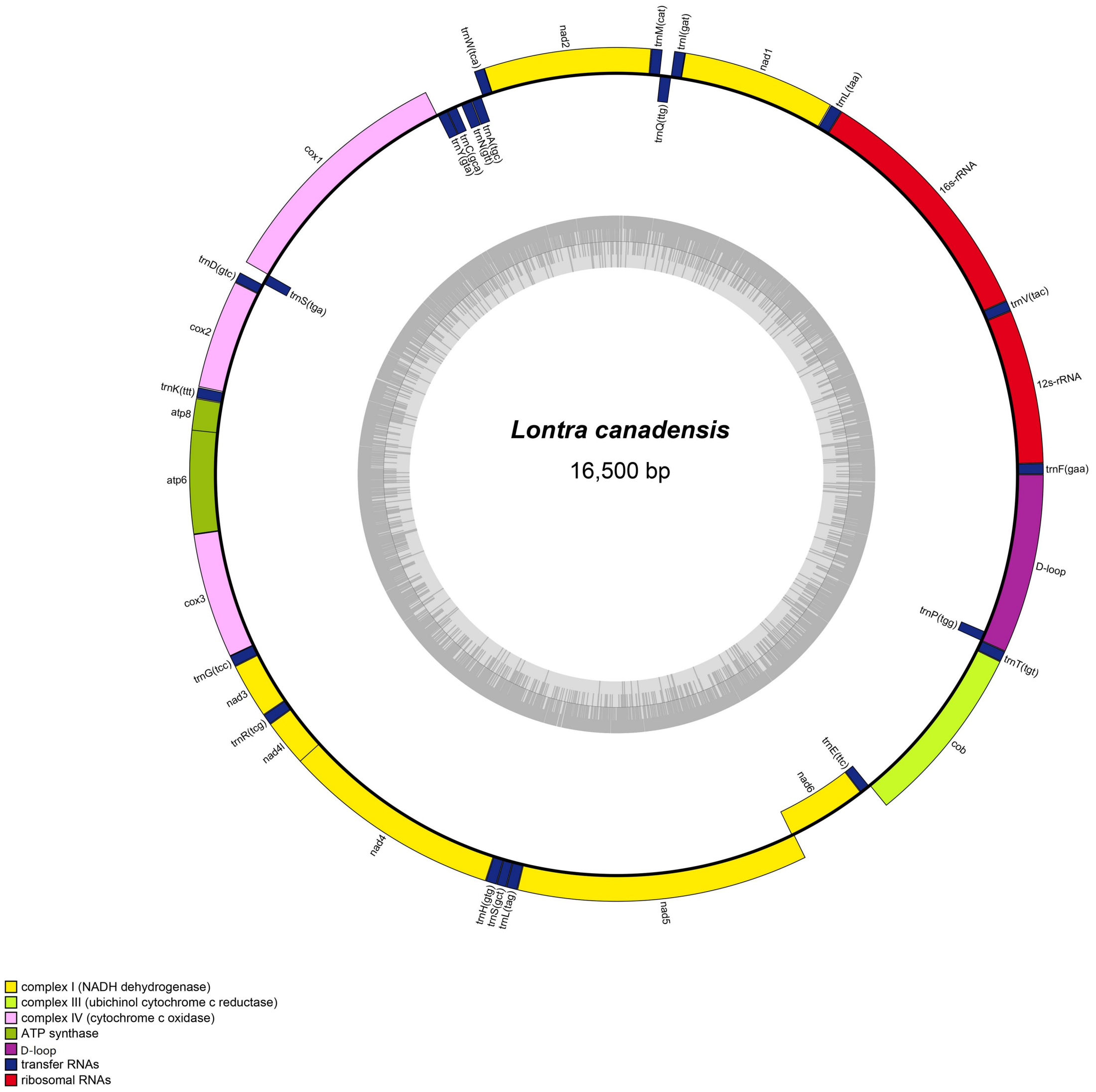

Figure S2. Mitochondrial genome structure map of *L. canadensis*.

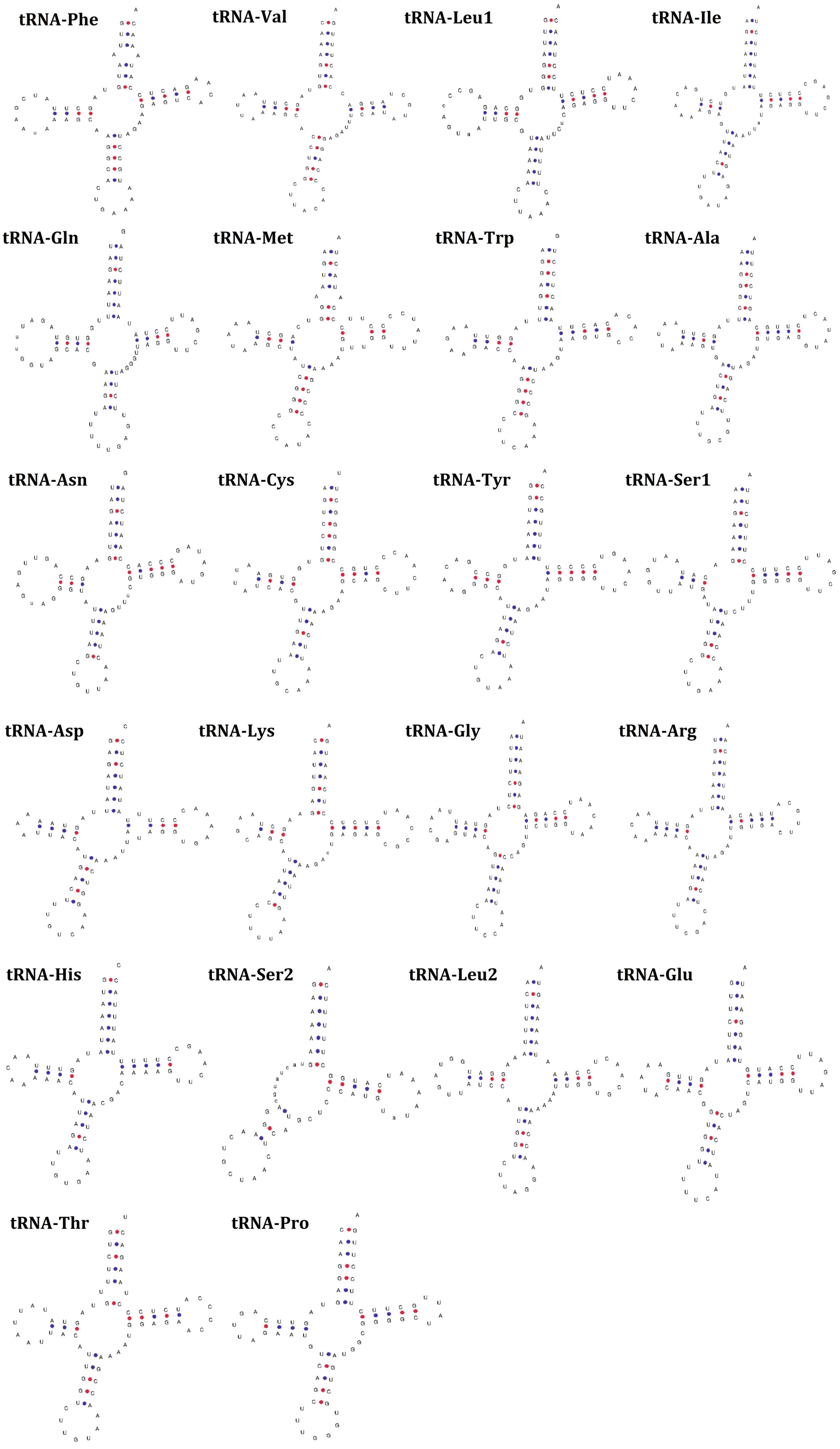

Figure S3. The predicted secondary structures of 22 tRNAs genes in *L. canadensis* Mitochondrial genome.
